# Supplementary material for: Mixed synapses reconcile violations of the size principle in zebrafish spinal cord
Source: eLife. 2022 Sep 27;11:e64063. doi: 10.7554/eLife.64063 (PMC9514842; doi:10.7554/eLife.64063)
Supplement: Transparent reporting form [file elife-64063-transrepform1.docx]

***eLife’s* transparent reporting form**

We encourage authors to provide detailed information *within their submission* to facilitate the interpretation and replication of experiments. Authors can upload supporting documentation to indicate the use of appropriate reporting guidelines for health-related research (see [EQUATOR Network](http://www.equator-network.org/%20)), life science research (see the [BioSharing Information Resource](https://biosharing.org/)), or the [ARRIVE guidelines](http://www.plosbiology.org/article/info:doi/10.1371/journal.pbio.1000412) for reporting work involving animal research. Where applicable, authors should refer to any relevant reporting standards documents in this form.

If you have any questions, please consult our Journal Policies and/or contact us: [editorial@elifesciences.org](mailto:editorial@elifesciences.org).

**Sample-size estimation**

- You should state whether an appropriate sample size was computed when the study was being designed
- You should state the statistical method of sample size computation and any required assumptions
- If no explicit power analysis was used, you should describe how you decided what sample (replicate) size (number) to use

Please outline where this information can be found within the submission (e.g., sections or figure legends), or explain why this information doesn’t apply to your submission:

Sample sizes were based on counts of numbers per spinal segment from transgenic lines. Estimations per spinal segment were used to determine a reasonable number of neurons to appropriate sample the population (twice the number per spinal segment). It was not possible to anticipate variance or choose specific numbers in advance because analogous work had not been done and we had not a priori knowledge of potential heterogeneity. For the purposes of this study, data had already been collected during previous studies of spinal motor neurons and different interneuron populations. An explanation of the source data and the numbers of new recordings included in the new analysis are reported at the beginning of the Results and Discussion section. Numbers are also reported with their respective graphs in the Figures and the Figure Legends.

**Replicates**

- You should report how often each experiment was performed
- You should include a definition of biological versus technical replication
- The data obtained should be provided and sufficient information should be provided to indicate the number of independent biological and/or technical replicates
- If you encountered any outliers, you should describe how these were handled
- Criteria for exclusion/inclusion of data should be clearly stated
- High-throughput sequence data should be uploaded before submission, with a private link for reviewers provided (these are available from both GEO and ArrayExpress)

Please outline where this information can be found within the submission (e.g., sections or figure legends), or explain why this information doesn’t apply to your submission:

How often each experiment is performed is associated with the relevant text or legend. We have only reported biological replication (of neurons), technical replication is not relevant here. All N values have been reported in appropriate locations in the manuscript. No outliers were excluded from analysis and individual data points are all included in graphs. Quantitative and qualitative criteria for inclusion (primarily related to neuron health) are stated in the Methods section (e.g., resting membrane potential). High-throughput sequence data are not included in this study.

**Statistical reporting**

- Statistical analysis methods should be described and justified
- Raw data should be presented in figures whenever informative to do so (typically when N per group is less than 10)
- For each experiment, you should identify the statistical tests used, exact values of N, definitions of center, methods of multiple test correction, and dispersion and precision measures (e.g., mean, median, SD, SEM, confidence intervals; and, for the major substantive results, a measure of effect size (e.g., Pearson's r, Cohen's d)
- Report exact p-values wherever possible alongside the summary statistics and 95% confidence intervals. These should be reported for all key questions and not only when the p-value is less than 0.05.

Please outline where this information can be found within the submission (e.g., sections or figure legends), or explain why this information doesn’t apply to your submission:

Statistical methods are given and explained in the last paragraph of the Material and Methods section. Statistical tests and their relevant parameters are listed in the relevant figure legends. Raw data (traces and values for all neurons in a population) are included for each experiment, where appropriate. Exact p values are reported, except for p < 0.001.

(For large datasets, or papers with a very large number of statistical tests, you may upload a single table file with tests, Ns, etc., with reference to sections in the manuscript.)

**Group allocation**

- Indicate how samples were allocated into experimental groups (in the case of clinical studies, please specify allocation to treatment method); if randomization was used, please also state if restricted randomization was applied
- Indicate if masking was used during group allocation, data collection and/or data analysis

Please outline where this information can be found within the submission (e.g., sections or figure legends), or explain why this information doesn’t apply to your submission:

Data were organized based on neuronal identity (e.g., motor neurons vs distinct types of interneurons) or based on input resistance. Masking was not used.

**Additional data files (“source data”)**

- We encourage you to upload relevant additional data files, such as numerical data that are represented as a graph in a figure, or as a summary table
- Where provided, these should be in the most useful format, and they can be uploaded as “Source data” files linked to a main figure or table
- Include model definition files including the full list of parameters used
- Include code used for data analysis (e.g., R, MatLab)
- Avoid stating that data files are “available upon request”

Please indicate the figures or tables for which source data files have been provided:

Source data files have been provided for Figures 1-3 (Figure 1- source data 1, Figure 2- source data 1, Figure 3- source data 1).
